# Case report: Cerebellar swelling and hydrocephalus in familial hemophagocytic lymphohistiocytosis

**DOI:** 10.3389/fped.2022.1051623

**Published:** 2022-12-16

**Authors:** Taro Yoshida, Kunihiko Moriya, Keisuke Oikawa, Shoko Miura, Yoshiko Asakura, Sachiko Tanifuji, Shuji Kusano, Mikiya Endo, Manami Akasaka

**Affiliations:** ^1^ Department of Pediatrics, School of Medicine, Iwate Medical University, Iwate, Japan; ^2^ Department of Pediatrics, Tohoku University Graduate School of Medicine, Sendai, Japan

**Keywords:** cerebellar swelling, hydrocephalus, familial hemophagocytic lymphohistiocytosis, inborn errors of immunity, newborn screening (NS)

## Abstract

Familial hemophagocytic lymphohistiocytosis (FHL) is a severe inborn error of immunity caused by a genetic defect that impairs the function of cytotoxic T and NK cells. There are only a few reported cases of FHL with diffuse swelling of the cerebellum and obstructive hydrocephalus. We report a case of FHL3 with neurological symptoms associated with cerebellar swelling and obstructive hydrocephalus. A male patient was hospitalized several times due to fever and decreased feeding, hepatosplenomegaly, and cytopenia since the first month of life. At 7 months of age, disturbance of consciousness was seen. Brain magnetic resonance imaging revealed signal intensity in the bilateral cerebellar hemispheres, diffusely increased periventricular white matter, and ventriculomegaly. Although he was treated with methylprednisolone pulse therapy, he was unresponsive to the treatment. He was then transferred to a local hospital after tracheotomy but died. Targeted clinical sequencing revealed a homozygous splice-site mutation in *UNC13D*. Pediatric hemophagocytic lymphohistiocytosis (HLH) includes some cases of central nervous symptom (CNS)-isolated HLH or CNS HLH preceding systemic lesions, which often do not initially meet the diagnostic criteria for FHL. Patients with FHL initiated by cerebellar symptoms may present with an atypical clinical course for HLH, leading to delayed diagnosis and poor outcomes. Despite the usefulness of a combination of a high percentage of lymphocytes in the peripheral leukocytes, a low lactate dehydrogenase level, and a high sIL-2R/ferritin ratio for identifying FHL, the diagnosis may be missed due to the absence of these results. Presymptomatic diagnosis of FHL by screening of newborns and subsequent early treatment of patients with a predicted poor prognosis may contribute to better outcomes.

## Introduction

Familial hemophagocytic lymphohistiocytosis (FHL) is a severe inborn error of immunity caused by a genetic defect that impairs the function of cytotoxic T and natural killer (NK) cells (1). The clinical symptoms of patients with FHL may vary widely. Patients with FHL may present with a variety of neurological symptoms prior to diagnosis, which in some cases can be fatal (2). The prognosis of patients with FHL has improved recently, and they can be cured with immunochemotherapy and hematopoietic stem cell transplantation (3). The diagnosis is difficult in some cases because the diagnostic criteria are not fulfilled at the initial presentation in some cases (4). Nonspecific symptoms in the early phase may delay the diagnosis of FHL. Early diagnosis through awareness of neurological symptoms may contribute to appropriate treatment and improved prognosis. Although some cases of FHL with diffuse swelling of the cerebellum and associated progression of obstructive hydrocephalus have been reported (5–8), it is still not well recognized among clinicians. It is a rare but life-threatening condition in which obstructive hydrocephalus occurs as a result of cerebellar swelling. We report a case of FHL3 with neurological symptoms associated with diffuse cerebellar swelling and obstructive hydrocephalus.

## Case descriptions

**Figure 1:**
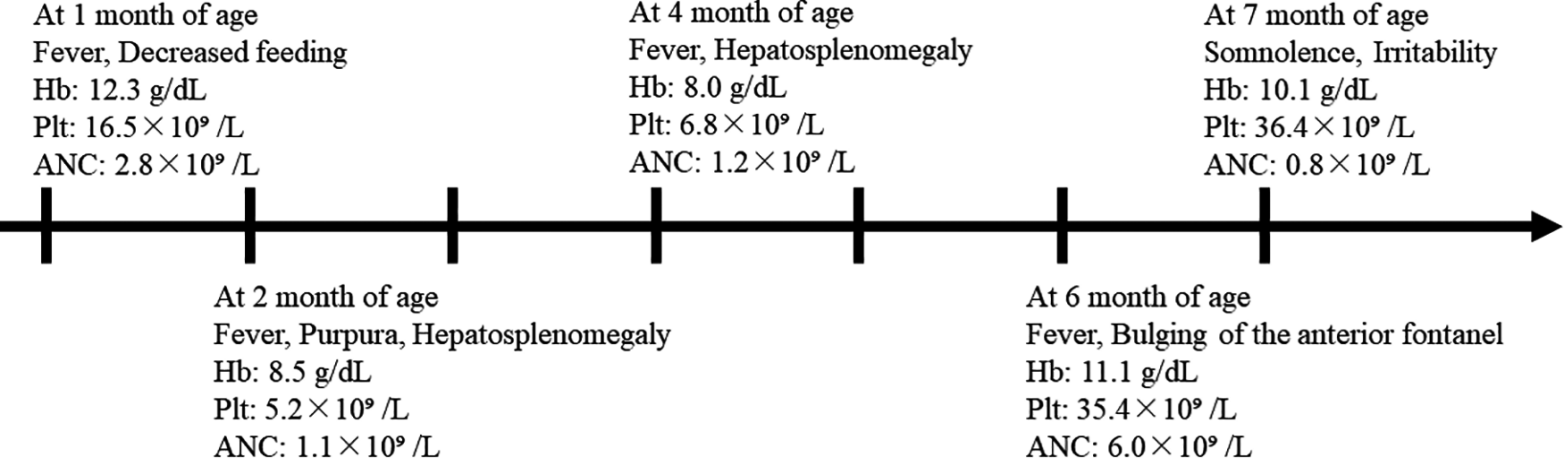
Clinical course and peripheral blood counts of our case. The horizontal axis shows the time course of this case, with one scale representing 1 month. Hb, hemoglobin; Plt, platelet count; ANC, absolute neutrophil count.

The patient was the second child of healthy, nonconsanguineous Japanese parents, and his sister was in good health at the time. He was born at 37 weeks of gestation, weighing 3,064 g. His perinatal course was uneventful. We show the clinical course of our case in Figure 1. At 1 month of age, he was brought to a local hospital due to a high fever and decreased feeding, which was managed with antimicrobials as clinicians suspected the presence of an infection. Although he developed transient bicytopenia, he was discharged. At 2 months of age, he was admitted for the second time due to fever, purpura, hepatosplenomegaly, and decreased hemoglobin level and platelet count. Laboratory findings showed elevated lactate dehydrogenase (LDH) and ferritin levels and increased atypical lymphocytes, while bone marrow examination showed no hemophagocytosis. He was treated with intravenous immunoglobulins and antimicrobials. At 4 months of age, the patient was admitted for the third time due to fever and hepatosplenomegaly and decreased hemoglobin level and platelet count. The results of a repeat bone marrow examination were normal. He was treated with cyclosporine and prednisolone. Oral sulphamethoxazole/trimethoprim was also initiated. The patient was admitted for the fourth time at 6 months of age due to fever and bulging of the anterior fontanel. Spinal fluid tests demonstrated normal findings; thus, he was discharged with antimicrobial treatment to relieve the fever.

**Figure 2:**
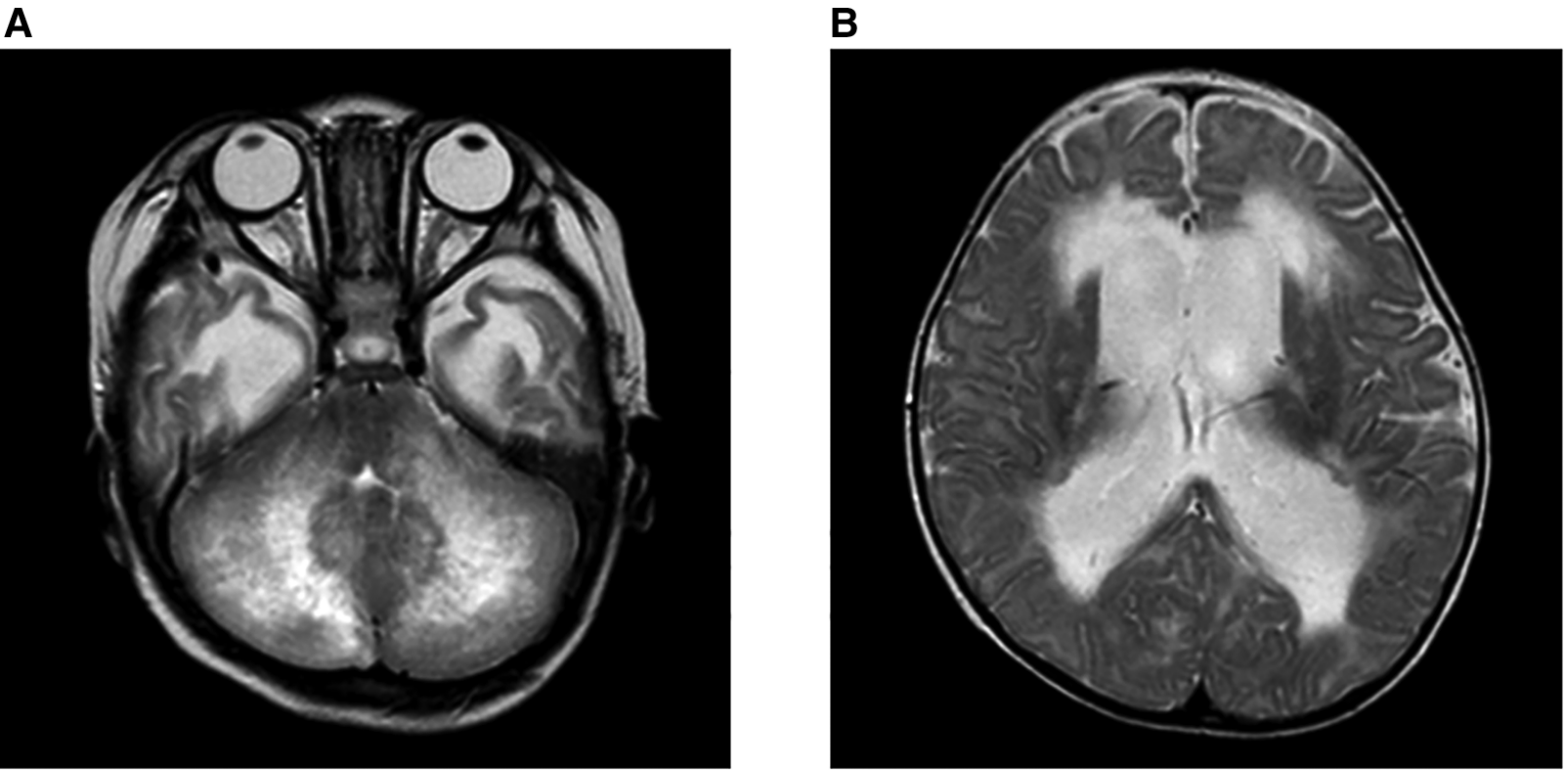
Brain MRI on admission at 7 months of age. Brain MRI on the 10 days after onset, on the T2WI. (**A**) Signal intensity of the bilateral cerebellar hemispheres and periventricular white matter were diffusely increased. (**B**) Ventriculomegaly due to the fourth ventricle obstruction. The diffuse cerebellar swelling caused the obstructive hydrocephalus.

At 7 months of age, he was seen for somnolence and irritability. Brain magnetic resonance imaging (MRI) revealed cerebellar swelling and hydrocephalus; thus, the patient was referred to a university hospital and was admitted for the fifth time. His weight and height were 7.3 kg [−1.3 standard deviation (SD)] and 71 cm (+0.2 SD), respectively. His anterior fontanel was bulging. His irritability was observed when his head was moved. He could not fix his eyes or perform light tracking. His head control was unstable. His direct light reflex on the left side was prompt, but that on his right side was sluggish. He showed facial nerve palsy on the left side. He was able to perform antigravity movements of his limbs, and his deep tendon reflexes were normal. Table 1 shows the clinical and laboratory findings on admission. Patients fulfilled three out of eight of the HLH diagnostic criteria (hypofibrinogenemia, increased ferritin, and elevated sIL-2R). T2WI brain MRI at 10 days after onset showed the presence of signal intensity of the bilateral cerebellar hemispheres and diffusely increased periventricular white matter. Ventriculomegaly due to fourth ventricle obstruction was also noted. The diffuse cerebellar swelling caused the obstructive hydrocephalus (Figure 2). Cerebrospinal fluid examination was not performed. Other laboratory tests showed no remarkable cytopenia and scant elevated liver enzymes and LDH levels. Epstein–Barr virus (EBV) viral capsid antigen (VCA) IgG, VCA IgM, and EBV nuclear antigen were negative, and neither IgG nor IgM of human herpes virus 6 and 7 was found. Rapid viral antigen tests were negative for the respiratory syncytial virus, mycoplasma, and influenza. Blood cultures were also negative.

**Table 1:** Patient’s clinical and laboratory findings and HLH diagnostic criteria.

	Patient findings	HLH-2004 diagnostic criteria (at least five out of eight main features)
Fever	36.9°C	≥38.5°C, more than 7 days
Hepatosplenomegaly	No	Radiographic or physical exam evidence
Cytopenia		2 or 3 hematopoietic lineages
Hemoglobin, g/dl	10.1	<9
Platelets, 10^9^/L	364	<100
Neutrophils, 10^9^/L	0.8	<1.0
AST, U/L	31	
ALT, U/L	10	
LDH, U/L	248	
Bilirubin, mg/dl	0.5	
Triglycerides, mg/dl	92	≥265 and/or
Fibrinogen, mg/dl	214	≤150
Ferritin, ng/ml	3,560	≥500
sIL-2R, U/ml	3,560	>2,400
Hemophagocytosis	No	Present in bone marrow or others
Decreased NK cell activity	ND	According to the local laboratory reference

HLH, hemophagocytic lymphohistiocytosis; AST, aspartate aminotransferase; ALT, alanine aminotransferase; LDH, lactate dehydrogenase; sIL-2R, soluble interleukin-2 receptor; NK, natural killer; CSF, cerebrospinal fluid; ND, not described.

We consulted with the neurosurgeon and ruled out osteotomy of the posterior cranium; thus, we proceeded with conservative treatment. We administered osmotic diuretics and dexamethasone with the expectation of a cerebral pressure-reducing effect. On the first day of admission, as there were rhythmic movements of the right limbs and electroencephalography (EEG) showed diffuse slow waves, we judged that the patient must be having a seizure; thus, we administered fosphenytoin. On the third day of admission, the patient developed a fever and appeared to be mouthing; hence, we administered a continuous dose of midazolam, which resulted in a decrease in blood pressure, and dopamine was also administered. On day 4 of hospitalization, the patient suddenly had ventricular fibrillation and cardiac arrest; a computed tomography scan showed cerebral herniation. After resuscitation, the patient was treated with methylprednisolone pulse therapy based on the belief that the brain herniation was caused by cerebral edema due to high cytokine levels. His blood pressure was subsequently stabilized. We continued to administer prednisolone. The patient had central enuresis and hypothyroidism. EEG results and auditory brainstem response were negative, and after a discussion with his parents, he was transferred to a local hospital after a tracheotomy, where he subsequently died. Targeted clinical sequencing of 12 genes known to cause FHL (The Twist BioScience custom targeted panel, Illumina NextSeq) was performed on the blood samples, which revealed mutation c.754-1G > C at the homozygous status in *UNC13D*, leading to the diagnosis of FHL.

## Discussion

This report presents a case of an infant who presented with CNS symptoms after multiple episodes of fever and cytopenia. Our patient presented with neurological symptoms due to cerebellar swelling and associated hydrocephalus. Pediatric FHL includes some cases of CNS-isolated HLH or CNS HLH preceding systemic lesions, which often do not initially meet the diagnostic criteria for FHL (9). Brain MRI findings in HLH are heterogeneous, with multiple white matter lesions being the most common (66%), followed by cerebellitis (19%) and brainstem dominant disease (15%) (9). Some HLH patients with cerebellitis have neurological symptoms preceding the onset of systemic HLH, and their diagnostic laboratory findings are negative. Patients with FHL initiated by cerebellar symptoms may present with an atypical clinical course for HLH, leading to delayed diagnosis and poor outcomes. We summarized a literature cohort of four cases of FHL with cerebellar swelling in Table 2. The cases included three boys and one girl: two with *PRF1* mutations and one each with other *UNC13D* and *STX11* mutations. In some cases, cerebellar swelling appears early and ahead of other cases, whereas, in others, as in our case, symptoms due to cerebellar swelling appear over time. Of the five cases, only two showed significant hemophagocytosis in the bone marrow. All cases except ours reached hematopoietic stem cell transplantation and survived, and three cases had no neurological complications.

**Table 2:** Clinical and laboratory findings in patients diagnosed with FHL complicated by cerebellar swelling.

	Case 1	Case 2	Case 3	Case 4	Our case
Ethnical origin	Unknown	Unknown	Unknown	Saudi	Japan
Familial disease	Two maternal uncles had died at ages 10 and 16 years	No	No	Unknown	No
Parental consanguinity	No	No	No	ND	No
Sex	Male	Female	Male	Male	Male
Type of FHL	2	2	3	4	3
Mutated gene	PRF1	PRF1	UNC13D	STX11	UNC13D
Allele 1	p. Leu215Ile	c.273C > T	p. Ile712_Gly713_delinsSer	p. Leu58Pro	c.754-1G > C
Allele 2	p. Ala262Asn	c.273C > T	p. Arg782Serfs * 12	p. Leu58Pro	c.754-1G > C
Age at diagnosis of HLH, months	36	156	84	31	7
Symptoms at diagnosis	Fever, hepatosplenomegaly	Headache, vomiting	Fever, hepatosplenomegaly	Fever, vomiting	Feeding disorder
Neurologic manifestations^a^	Ataxia, nystagmus, dysmetria, coma, epilepticus	Gait imbalance, double vision	Unsteady gait, ataxia, dysmetria, dysdiadochokinesia, dysarthria	Irritability, ataxic gait, somnolence	Somnolence, irritability
WBC, µl	ND	4,840	ND	5,320	3,980
Lymphocyte, %	ND	55	ND	ND	75
AST, U/L	60	39	ND	208	31
ALT, U/L	ND	38	ND	228	10
LDH, U/L	ND	ND	ND	ND	248
Triglycerides, mg/dl	213	ND	ND	319	92
Ferritin, ng/ml	179	ND	ND	2,578	11,866
sIL-2R, U/ml	ND	ND	ND	ND	3,560
Hemophagocytosis	Occasionally	rare	Yes	No	No
Findings of Cerebrospinal fluid	Pleocytosis (61 cell/mm^3^)	High levels of IgG and IgM	ND	Normal	ND
Findings of brain MRI	Cerebellar swelling, downward herniation of cerebellar tonsils	Swollen white matter of the cerebellum extending into the axis in the T2-weighted image, resulting in tonsillar herniation	Hyperintense signal changes in cortex and white matter of cerebellar hemispheres and diffuse cerebellar edema in T2 weighted image	Diffuse enlargement of both cerebellar hemispheres, Mass effect on the fourth ventricle and brainstem, Mild herniation of cerebellar tonsils	Diffuse enlargement of both cerebellar hemispheres, hydrocephalus
Pre-transplant treatment	HLH-94	HLH-2004	Chemotherapy	HLH-2004	mPSL
Age at HSCT, months	48	459	ND	35	Not done
Conditioning regimen	Bu + CY + VP − 16	ND	ND	Flu + L − PAM + ATG	–
Outcome	Alive	Alive	Alive	Alive	Dead
Neurological complications	Seizure, behavior disturbances, hypoacusia, visual field defects	None	None	None	–
Observation period	16 months	18 months	ND	9 months	8 months
Reference	(7)	(8)	(9)	(10)	This study

FHL, familial hemophagocytic lymphohistiocytosis; HLH, hemophagocytic lymphohistiocytosis; WBC, white blood cell count; AST, aspartate aminotransferase; ALT, alanine aminotransferase; LDH, lactate dehydrogenase; sIL-2R, soluble interleukin-2 receptor; HSCT, hematopoietic stem cell transplantation; Bu, busulfan; CY, cyclophosphamide; VP-16, etoposide; L-PAM, melphalan; ATG, anti-thymocyte globulin; ND, not described.

^a^ Reported at some point during the course of the disease.

Our patient died before we reached a definitive diagnosis, despite having had several opportunities for inpatient treatment. We have performed tests, including bone marrow examination, but have not obtained definitive evidence of disease. We had initially considered a possible diagnosis of autoimmune lymphoproliferative syndrome because of the patient's hepatosplenomegaly, a course of multiple cytopenias, and an elevated double negative T cell at 9.6% (10). Despite examinations for possible differential diseases as possible causes of cerebellar swelling, such as viral infections (rotavirus, herpesvirus, mycoplasma), congenital metabolic abnormalities, and drug-induced neurological disorders, all of these had negative findings. Patients had no family history of immunodeficiency and did not have an autoimmune disease phenotype. Contrarily, he had only two positive findings (ferritin and sIL-2R) according to the HLH criteria (11) when the patient presented for cerebellar symptoms. These two positive findings were determined using samples collected during resuscitation for cardiac arrest at 4 days after admission. The patient also met up to three of the HLH criteria at other admissions (fever, hepatosplenomegaly, and cytopenia) and did not fully meet the HLH criteria at any time point. Yasumi et al. have indicated the usefulness of a combination of a high percentage of lymphocytes in peripheral leukocytes (74.0% ± 14.4%), low levels of LDH (489 ± 163 IU/L), and a high ratio of sIL-2R to ferritin (13,500 ± 12,800 IU/µg) for identifying FHL (12). The patient had a high lymphocyte percentage and relatively low LDH levels (all data not shown) on all of his several admissions and a low sIL-2R/ferritin ratio on his first admission. The sIL-2R/ferritin ratio was not measured at any other admission. These markers may be useful in predicting FHL; however, the diagnosis may be missed due to some cases not being matched to them. Our case indicates that for patients presenting with unexplained multiple recurrent fevers and cytopenia, it is necessary to actively consider the possibility of inborn errors of immunity, including HLH, and perform functional tests, which can lead to an early diagnosis. The incidence of FHL was estimated as 1 in 50,000–300,000 live births in Japan (13). FHL is a candidate for newborn screening (NS) because it is a life-threatening condition after onset, and early diagnosis and curative treatment can improve its prognosis. Indeed, the polymerase chain reaction-based NS has been reported for FHL3 by *UNC13D* inversion (14). Although further prospective evaluation in NS for FHL may be needed, presymptomatic diagnosis of FHL by NS and subsequent early treatment of patients with a predicted poor prognosis may contribute to better outcomes.

## Data Availability

The raw data supporting the conclusions of this article will be made available by the authors without undue reservation.
